# Active and sham transcranial direct current stimulation (tDCS) improved quality of life in female patients with fibromyalgia

**DOI:** 10.1007/s11136-022-03106-1

**Published:** 2022-03-01

**Authors:** N. Samartin-Veiga, A. J. González-Villar, M. Pidal-Miranda, A. Vázquez-Millán, M. T. Carrillo-de-la-Peña

**Affiliations:** 1 Brain and Pain (BaP) Lab, Departamento de Psicoloxía Clínica y Psicobioloxía, Facultade de Psicoloxia, Universidade de Santiago de Compostela, Campus Vida, 15782 Santiago de Compostela, A Coruña, Spain; 2 Psychological Neuroscience Lab, Research Center in Psychology, School of Psychology, University of Minho, Braga, Portugal

**Keywords:** Fibromyalgia, Transcranial direct current stimulation (tDCS), Quality of life (QoL), SF-36, FIQ-R, Randomized controlled trial (RCT)

## Abstract

**Purpose:**

Fibromyalgia (FM) is a chronic pain syndrome with a strong impact on quality of life (QoL). Treatment of this condition remains a challenge, due to the scarce evidence for the effectiveness of the therapeutic approaches available. Current attention is focused on transcranial direct current stimulation (tDCS), which has yielded promising results for pain treatment. Rather than focusing only on pain relief, in this study, we aimed to determine how active or sham tDCS (over three cortical targets -the primary motor cortex, the dorsolateral prefrontal cortex and the operculo-insular cortex-) affect QoL in patients with FM.

**Methods:**

Using a double-blind, placebo-controlled design, we applied fifteen tDCS sessions of 20’ to initial 130 participants (randomized to any of the four treatment groups). We evaluated the QoL (assessed by SF-36) and the symptoms’ impact (assessed by FIQ-R) in baseline, after treatment and at 6 months follow-up.

**Results:**

All groups were comparable as regards age, medication pattern and severity of symptoms before the treatment. We found that QoL and symptoms’ impact improved in all treatment groups (including the sham) and this improvement lasted for up to 6 months. However, we did not observe any group effect nor group*treatment interaction.

**Conclusions:**

After the intervention, we observed a non-specific effect that may be due to placebo, favoured by the expectations of tDCS efficacy and psychosocial variables inherent to the intervention (daily relationship with therapists and other patients in the clinic). Therefore, active tDCS is not superior to sham stimulation in improving QoL in FM.

**Supplementary Information:**

The online version contains supplementary material available at 10.1007/s11136-022-03106-1.

## Introduction

Fibromyalgia (FM) is a chronic pain syndrome that affects between 0.4 and 11% of the population (Wolfe et al., 2018); mainly women (80–90% of the diagnosis) [1–3]. FM is characterized by widespread musculoskeletal pain, fatigue, sleep and mood disorders, and cognitive impairment [4]. These enduring symptoms can result in impaired health-related Quality of Life (QoL); in fact, several studies have consistently reported low QoL in patients with FM, with effects on physical, psychological and social domains [5–7]. Specifically, the aspects that most condition QoL in FM are physical problems (pain mainly) [8], social support, emotional status, educational level and age [8–10]. The large prevalence of FM the persistence of symptoms and the associated poor QoL result in high direct (medical costs) and indirect expenses (e.g. sick leave or disability pension) [11].

Treatment of FM remains a challenge. Current clinical guidelines for the management of this syndrome suggest a multidisciplinary approach, including pharmacotherapy, therapeutic exercise, patient education and cognitive-behavioural therapy [12, 13]. However, these therapies usually only provide moderate relief of FM symptoms [13, 14]. Although the aetiology of FM is unknown, it is assumed that central sensitization and impaired endogenous modulation of pain are important factors [15–17]. Transcranial Direct Current Stimulation (tDCS), a non-invasive neuromodulation technique, has been used to modify maladaptive brain mechanisms related to pain chronification [18]. During tDCS, a low-intensity electrical current (0.5–2.0 mA) is delivered through electrodes placed on the scalp [19]. The technique has been applied mainly over the primary motor cortex (M1) in FM patients, resulting predominantly in pain relief [20]. The tDCS has been awarded an A level of recommendation (i.e. established as effective) for the clinical treatment of pain [21]. However, most of these studies present some methodological flaws (i.e. small sample size, lack of a placebo group or double-blind control), and a large heterogeneity in the stimulation protocols (variations in the number of sessions, in the intensity of current, or in the cortical target) [20, 22]. Moreover, although tDCS can induce lasting changes at the synaptic level through long-term potentiation (LTP) mechanisms [23–25], evidence on the long-term effects is limited by the absence of follow-up assessments in many studies [18, 26]. Moreover, knowledge about the neurophysiological mechanisms underlying the effects of tDCS over M1 or about the optimal cortical target is limited. It has been proposed that tDCS over M1 modulate M1-thalamic inhibitory networks [27] and the M1 projections with cortical and subcortical nociceptive regions [27, 28]; however, more evidence is needed to clarify the specific mechanisms. This technique has also been applied over the dorsolateral prefrontal cortex (DLPFC), yielding improvement in cognitive and affective symptoms of patients with FM [29–31]. Stimulation over the DLPFC could decrease fronto-thalamic connectivity [32] and possibly influence nociceptive descending modulation mechanisms [33], given its connections with the anterior cingulate cortex, insula and subcortical structures. Although the exploration of other cortical areas specifically involved in pain processing would be of interest, so far this has not been investigated. The operculo-insular cortex (OIC) plays a special role in modulating the emotional aspects of pain [34], given its connections with the thalamic, limbic and multisensory cortices [35, 36]. FM, neuroimaging studies showed a decreased in grey matter volume in the insular cortex [37], and hypoactivation of the inferior parietal cortex [38]. Thus, exploration of the effects of tDCS over the OIC for the relief of FM symptoms, especially pain, is of great interest.

Most previous tDCS trials have focused on the efficacy of treatment for specific symptoms, such as pain [39], and not on the overall health status of patients with FM. Given the strong impact of FM on QoL and the recommendation of treatment guidelines to use QoL as the primary treatment outcome [40, 41], randomized clinical trials should be conducted to assess the effect of tDCS on patients’ QoL and on symptoms’ impact on it [42]. In addition, although a statistically significant change in any outcome variable may not have real clinical impact [43], previous studies have scarcely included analysis of the minimal clinically important difference (MCID). It has been reported that the improvement of pain in FM after tDCS is superior to the MCID [20], but to our knowledge, no studies have evaluated the MCID on quality of life scores.

In order to address these knowledge gaps, the main objective of the present clinical trial was to assess the effectiveness of tDCS on the QoL of patients with FM. We considered different dimensions of QoL (assessed by the SF-36 questionnaire) and, also, the impact of the disease on everyday functioning (assessed by the Fibromyalgia Impact Questionnaire, FIQ-R). To this end, we performed a double-blind, randomized, placebo-controlled trial, applying tDCS during 3 weeks, to a sample of 130 patients with FM. Additional objectives were to assess the long-term effects of the treatment (6-month follow-up), to determine the optimal tDCS target (comparing active stimulation over M1, DLPFC and OIC and a sham condition) and, to study if the improvement after tDCS was clinically important. We expected active tDCS to have a greater effect on QoL than that produced by the sham stimulation. Given the great influence of pain on QoL [8], we also hypothesized that the tDCS effects would be superior when a more specific pain area such as the OIC is targeted. Moreover, we assumed that the clinical improvement produced by active stimulation would last longer (up to 6 months) than any improvement generated by sham stimulation.

### Methods

#### Participants

The study initially included 132 women diagnosed with FM. All participants were aged between 25 and 65 years and had a previous diagnosis of fibromyalgia, in accordance with the American College of Rheumatology (ARC) criteria of 2010 [44]. The following exclusion criteria were applied: immune system pathology or comorbidities that could explain the main symptomatology; history of substance abuse; diagnosis of psychiatric diseases (except depression and anxiety); presence of brain damage or neurodegenerative disease; risk factors for the tDCS procedure (history of epilepsy); and the use of drugs with effects on sodium and calcium channels (e.g. carbamazepine and gabapentin) [45, 46]. The patients should also have had a stable medication pattern for at least 2 months before starting the treatment, and they were asked to maintain the pattern during the clinical trial.

We enrolled patients through local health centres, press and patients’ associations, and also contacted participants of previous studies conducted by our research team. The initial contact was made by telephone. When patients who fulfilled the inclusion and exclusion criteria agreed to participate in the study, we made an appointment for the pre-treatment clinical evaluation. All participants were required to sign an informed consent form.

#### Design

We carried out a randomized, sham-controlled, double-blind clinical trial, between May 2017 and November 2018, in Galicia (Spain). The current study is an extension of a pre-registered trial in http://www.encepp.eu/ (registration number: 24294) and published in Samartin-Veiga et al. [47]. The protocol was approved by the Research Ethics Committee of Galicia (code: 2014/488), according to the Declaration of Helsinki.

**Fig. 1:**
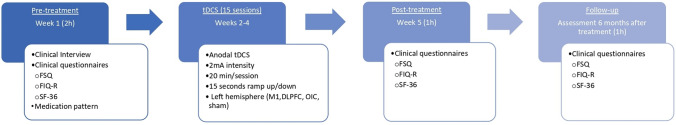
Overview of the study design at different time points (pre-treatment, treatment, post-treatment, and follow-up)

The study design is illustrated in Fig. 1. Before starting the clinical trial, we calculated the sample size based on previous literature in FM, where tDCS over M1 demonstrated a medium effect size [29, 48–53]. This effect size has been confirmed in a recent meta-analysis (Hedge's g = − 0.62) [54]. Using the program G*power (v 3.1.9.3) [55], we estimated that a minimum of 128 participants was needed to reach a small/medium effect size (*f* = 0.167) using a linear mixed model analysis of variance (ANOVA) (with three temporal assessments and four groups). We initially recruited 132 participants; 2 were excluded for not meeting the inclusion/exclusion criteria and the rest were randomly assigned to one of the four treatment groups (M1, DLPFC, OIC or Sham). The randomization protocol was performed by an independent experimenter using the order of entry into the study and a previous computer-generated randomization list (applying the ratio 1:1:1:1 for M1, DLPFC, OIC, Sham, to minimize the risk of generating unbalanced group sizes). Each participant was assigned an identification code related to a montage template, which contained the tDCS stimulation parameters (available in Neuroelectrics® software; NIC v.1.4.12). The researchers who performed the treatment only knew the code of each participant but could not visualize the template with the stimulation parameters. These researchers, blind to the condition (active/sham), also performed the statistical analyses.

The tDCS protocol was based on previous literature [30, 31, 48–50], and focused on achieving lasting effects through a greater number of sessions than earlier studies [31, 50]. Thus, the tDCS treatment consisted of 15 sessions, each of 20 min, administered along 3 weeks (Monday to Friday). The treatment was applied in several health centres or in the neuromodulation laboratory. Participants were permitted to miss a maximum of three treatment sessions.

## Procedure

### Clinical Evaluation (before, immediately after treatment and at follow-up).

In the pre-evaluation session, we conducted an interview to determine sociodemographic variables and the pattern of medication. The patients filled in the following questionnaires (all in their Spanish validated versions) to assess the severity of the symptoms, QoL and impact of the symptoms on the QoL:

The *Fibromyalgia Survey Questionnaire (FSQ)* [56, 57]. The FSQ includes the Widespread Pain Index (WPI) and the Symptom Severity Scale (SSS). WPI indicates the number of body areas where pain is experienced; its score ranges between 0 and 19 (where 0 indicates lack of painful areas and 19 that all areas are painful). The SSS assesses the level of tiredness/fatigue, non-restorative sleep, and cognitive problems, as well as abdominal pain, depression, and headache; its score ranges between 0 (no presence of these symptoms) and 12.

The *36-item Short Form Health Survey (SF-36)* [58, 59]. The SF-36 assesses QoL and provides a profile of health status and function. It is composed of 36 items distributed in 8 scales: physical function, physical role, body pain, general health, vitality, social function, emotional role, and mental health, i. e., the most relevant health concepts included in the Medical Outcomes Study (MOS). The scores on each subscale range from 0 to 100 (0 represents the worst possible health level and 100, the best). In this study, we calculated the score for the eight subscales and a mean score of the SF-36.

The *Fibromyalgia Impact Questionnaire revised (FIQ-R)* [60, 61]. This questionnaire assesses the impact of the FM symptoms on the functional capacity for daily living and work, as well as other aspects such as well-being, pain, anxiety, depression, morning stiffness and sleep quality. The FIQ-R includes 21 items (scored from 0 to 10) exploring three domains: physical functioning (30% of the score), general impact (20%) and severity of FM symptoms (50%). The maximum total score is 100 (corresponding to the highest severity/disability due to FM).

The assessment was performed before/after treatment and at a 6-month follow-up. The primary outcome variable considered in this research was the SF-36 mean score. We also analysed the effect of treatment on the individual SF-36 subscales, the FIQ-R total score and the 3 domains explored by the FIQ-R.

## tDCS

Participants were seated in a comfortable chair in a quiet room and instructed to remain at rest with their eyes open during the stimulation session (20 min.). To perform the tDCS, we used a Starstim tDCS device fixed with Velcro to the head cap, sponge electrodes dipped in saline solution, and Neuroelectrics® Information Controller software (NIC v.1.4.12) (Neuroelectrics®, Barcelona, Spain; http://neuroelectrics.com). Anodal tDCS stimulation was applied to three targets on the left hemisphere: M1, DLPFC and OIC. In each montage, the stimulation electrodes had a different location (following the International 10/10 System of electrode placement), shape, polarity, and intensity (2 mA). Specifically, to stimulate M1 and DLPFC, we used two-electrode montages (25 cm^2^ sponge disc electrodes) with the following parameters, respectively: C3 electrode = -2 mA and Fp2 electrode = 2 mA, and F3 electrode = -2 mA and Fp2 electrode = 2 mA. To stimulate the OIC, we used a multi-electrode montage (3.14 cm^2^ sponge disc electrodes) with the following parameters: F3 electrode = − 0.565 mA; FC1 electrode = − 0.508 mA; F8 electrode = − 0.158 mA; FC5 electrode = 0.579 mA; C5 electrode = 1.144 mA; and P3 electrode = − 0.492 mA (Bradley et al., in prep.). For the sham group, an independent experimenter assigned the participants to one of these three montages (M1, DLPFC or, OIC). The electrodes were placed in the corresponding cortical areas but without applying current during the sessions. This allocation remained constant throughout all tDCS sessions, maintaining the group assignment blind (active vs. sham). The ground electrode was located in the right earlobe. The caps were adjusted to the skull perimeter using different cap sizes (small, medium, large), to control the electrode placement.

In each session, the current intensity was ramped up and down. In the actively stimulated groups, a 15 s ramp-up was applied at the beginning of the stimulation period and, a 15 s ramp-down at the end of the session. In the sham group, ramps were applied up and down at the beginning and end of the sessions (15 s each), but no current was supplied during the interval between the initial and the final ramps.

## Statistical Analysis

We performed one-way ANOVAs to determine whether the treatment groups (M1, DLPFC, OIC and Sham) were comparable in age, clinical status (assessed by FSQ) and QoL (assessed by SF-36 and FIQ-R) before the treatment. Also, we used Chi-square analysis to test possible differences between the groups in the pattern of medication (previously classified into analgesics, non-steroidal anti-inflammatory drugs, anxiolytics, anti-epileptics, opioids, antimigraine, antidepressants, sedatives, antipsychotics, and other medication) and in the number of missed sessions (a maximum of three was allowed).

To follow an intention to treat (ITT) protocol, we included all randomized subjects and maintained their original assignment. For missing data, we previously modelled the outcome using mixed-effects regression (LMR) models, without imputation and with mean, median and Last Observation Carried Forward and Backward (LOCFB) imputations. The best linear fit, with the lowest Akaike criteria, was yielded by the model using the median imputation for SF-36 mean score and the mean imputation for the rest of the variables. More details about the LMR analysis can be found in Table 1 of the Supplementary Material. Then, we performed a two-tailed repeated measure ANOVA for each outcome variable (SF-36 subscales and mean; FIQ-R subscales and global scores), with Time (pre-treatment, post-treatment, and 6 months' follow-up) as a within-subject factor and Group (M1, DLPFC, OIC and Sham) as a between-subject factor. If any effect or interaction was found significant, we performed post hoc analysis (with Bonferroni-Holm correction for multiple comparisons). When appropriate, effect sizes (partial eta square; *ηp*^2^) are reported [62].

**Table 1:** Age and medication patterns of participants prior to starting treatment

	M1 (*n* = 32)	DLPFC (*n* = 33)	OIC (*n* = 33)	Sham (*n* = 29)	*F* (p)	Chi^2^ (*p*)
Age (mean ± SD)	49.38 ± 8.83	51.00 ± 9.15	50.21 ± 8.20	50.67 ± 8.88	0.21 (0.89)	–
*Medication*						
Analgesics	27.3%	38.2%	42.4%	31%	–	2.03 (0.57)
NSAIDS	45.5%	35.3%	36.4%	44.8%	–	3.25 (0.36)
Opioids	39.4%	26.5%	33.3%	31%	–	1.32 (0.73)
Antimigraine	0%	2.9%	3%	0%	–	1.88 (0.60)
Anxiolytics	51.5%	38.2%	63.6%	58.6%	–	4.86 (0.18)
Antidepressants	51.5%	41.2%	57.6%	65.5%	–	4.03 (0.26)
Sedatives	15.2%	11.8%	9.1%	6.9%	–	1.24 (0.75)
Antipsychotics	0%	5.9%	9.1%	3.4%	–	3.28 (0.35)

There were no differences between the treatment groups

To assess the minimal clinical important difference (MCID) after treatment and at follow-up, we calculated the percentages of improvement in the SF-36 and FIQ-R total scores (calculated for post-treatment as: (before minus after)/before × 100; and for follow-up as:(before minus follow-up)/before × 100). Moreover, for the total score of SF-36 and FIQ-R we performed one-way ANOVA analyses to test Group effects on the percentage of improvement in both time points (post-treatment and follow-up).

The LMR analyses were performed using the lme4 and emmeans package of R version 4.0.2 (The R Foundation) and statistical analyses were performed with SPSS (v. 25) with a significance level (*p*-value) of less than 0.05.

### Results

**Fig. 2:**
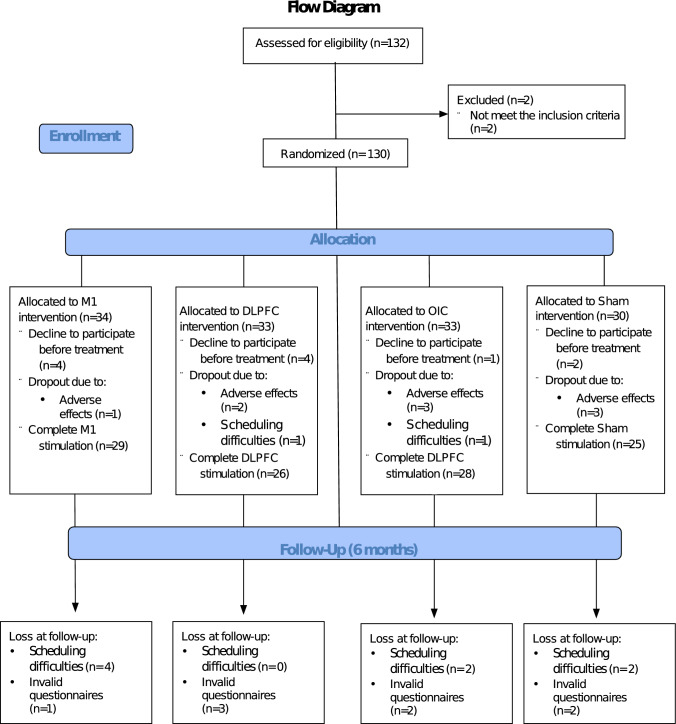
Flow diagram showing the number of participants and randomization (CONSORT model; 2010)

We contacted 132 participants, of whom 130 met the inclusion criteria and were allocated randomly to the 4 treatment groups. By groups, the number of participants were as follows: M1 (*n* = 34), DLPFC (*n* = 33), OIC (*n* = 33) and sham (*n* = 30). Eleven patients declined to participate before starting treatment, and another 11 dropped out once treatment began (9 for adverse effects and 2 for schedule issues). In the follow-up assessment, 8 participants did not properly complete the questionnaires and other 8 did not attend for schedule reasons. For the statistical analyses, following the ITT protocol, we used the 130 participants originally randomized, using the imputation for the missing values as explained above (see flow diagram in Fig. 2).

Before the treatment, we checked that the groups were matched in relation to age and education years. Also, they were comparable in age and medication patterns at baseline, as well as in severity of FM symptoms (assessed by FSQ: SSS; WPI), quality of life (assessed by SF-36) and impact on daily life (FIQ-R) (Tables 1 and 2). Finally, 108 participants completed the treatment. Since they were allowed to miss a maximum of three session, we tested possible group differences in the number of sessions missed, which were non-significant (Chi^2^ = 14.095; *p* = 0.723).

**Table 2:** Mean values of clinical variables at the pre-treatment, immediately post-treatment, and 6 months after treatment (Standard Deviation in parentheses); results of one-way ANOVAs for group effects before treatment

Group Variable	M1			DLPFC			OIC			Sham				Baseline difference		
	Pre mean (SD) *n* = 34	Post mean (SD) *n* = 29	6 months mean (SD) *n* = 24	Pre mean (SD) *n* = 33	Post mean (SD) *n* = 26	6 months mean (SD) *n* = 23	Pre mean (SD) *n* = 33	Post mean (SD) *n* = 28	6 months mean (SD) *n* = 24	Pre mean (SD) *n* = 30		Post mean (SD) *n* = 25	6 months mean (SD) *n* = 21	One-way ANOVA		
														*F*		*p*
FSQ	21.03 (4.55)	18.04 (6.40)	19.08 (6.38)	20.67 (5.18)	18.62 (6.72)	16.96 (6.53)	21.00 (4.72)	17.21 (6.84)	17.92 (6.52)	21.47 (5.64)		18.28 (5.81)	18.65 (6.50)	0.133		0.94
SSS	9.09 (1.92)	8.53 (2.51)	8.40 (2.47)	9.30 (2.39)	7.44 (2.50)	7.92 (2.58)	9.45(1.75)	7.25 (2.30)	8.04 (2.44)	8.93 (2.78)		7.64 (2.77)	8.17 (2.87)	0.332		0.80
WPI	11.93 (3.67)	9.50 (4.75)	11.13 (4.53)	11.36 (3.87)	10.89 (6.15)	9.40 (4.65)	11.54 (4.17)	9.96 (5.67)	9.88(4.71)	12.53 (4.44)		10.64 (4.48)	10.47 (4.96)	0.512		0.68
SF-36 Mean	34.91 (13.86)	42.13 (17.34)	42.43 (18.84)	37.76 (13.63)	45.15 (15.07)	46.78 (13.95)	35.69 (13.48)	44.22 (14.82)	46.09 (18.64)	32.96 (13.13)		41.35 (16.05)	40.86 (20.41)	0,679		0.57
Physical Function	43.13 (23.37)	42.93 (24.04)	48.12 (25.074)	44.854 (19.34)	46.92 (18.92)	55.83 (17.05)	40.61 (19.87)	43.39 (17.69)	47.29 (24.93)	40.50 (17.19)		45.40 (18.25)	50.45 (19.89)	0.313		0.82
Physical Role	26.95 (19.86)	35.48 (21.23)	41.41 (25.39)	33.33 (21.63)	35.27 (24.67)	39.84 (25.59)	26.51 (19.14)	42.00 (22.08)	45.31 (26.02)	25.42(21.06)		36.12 (26.02)	39.77 (29.54)	0.995		0.40
Body Pain	22.81 (14.44)	28.93 (20.64)	29.92 (23.13)	22.73 (15.50)	30.38 (20.76)	31.58 (16.62)	22.76 (16.68)	35.57 (19.68)	30.83 (18.15)	19.73 (16.18)		28.40 (17.35)	27.54 (20.90)	0.285		0.84
General Health	30.66 (18.66)	32.79 (21.02)	32.50 (20.16)	31.58 (17.80)	34.35 (19.39)	39.33 (16.93)	29.31 (14.58)	30.93 (13.73)	34.21 (17.61)	27.87 (16.18)		29.48 (15.74)	28.27 (18.64)	0.287		0.84
Vitality	23.91 (13.18)	30.17 (17.70)	31.46 (17.16)	26.52 (14.06)	32.69 (18.55)	33.75 (18.01)	26.36 (13.36)	33.04 (13.08)	34.17 (16.40)	23.66 (13.95)		30.20 (19.55)	29.32 (18.534)	0.404		0.75
Social Function	40.63 (21.53)	50.00 (30.07)	47.40 (28.55)	44.32 (25.79)	54.32 (25.97)	55.73 (21.17)	37.12 (25.67)	50.45 (20.83)	55.21 (28.77)	36.67 (22.97)		48.50 (27.56)	46.02 (30.47)	0.704		0.552
Mental Health	39.87 (15.82)	49.79 (17.59)	47.17 (18.69)	37.21 (15.51)	53.23 (17.66)	48.00 (17.01)	42.91 (13.87)	52.00 (16.47)	51.50 (15.84)	40.40 (14.57)		47.68 (17.81)	45.27 (20.45)	0.804		0.49
Emotional Role	51.30 (27.85)	66.95 (24.55)	61.46 (31.93)	61.87 (29.54)	74.04 (23.25)	70.14 (25.53)	54.04 (27.73)	66.37 (31.71)	71.18 (29.89)	49.44 (28.19)		65.00 (29.76)	60.23 (30.42)	1.198		0.31
FIQ-R total	70.65 (16.39)	56.27 (23.68)	56.15 (22.21	63.84(15.36)	50.73 (22.14)	54.69 (16.68)	68.01 (20.77)	49.99 (24.7)	54.46 (28.67)	67.68 (19.05)		53.75 (23.30)	54.80 (24.41)	0.774		0.51
FIQ-R Symptoms	37.63 (7.88)	29.12 (11.18)	29.96 (10.62)	35.27 (8.26)	26.50 (10.72)	30.50 (9.53)	35.51 (9.93)	25.41 (12.21)	29.98 (16.50)	36.98 (8.70)		27.66 (11.79)	30.70 (11.54)	0.544		0.65
FIQ-R Impact	13.18 (4.71)	10.97 (6.04)	10.12 (6.41)	11.03 (5.19)	9.54 (6.22)	8.54 (5.36)	12.72 (5.74)	9.00 (6.59)	9.31 (6.32)	12.57 (5.76)		10.08 (6.56)	9.77 (7.14)	0.996		0.40
FIQ-R Function	19.83 (5.79)	16.18 (8.33)	16.07 (7.22)	17.45 (6.01)	14.69 (7.69)	15.65 (6.04)	19.77 (6.61)	15.58 (7.61)	15.17 (8.17)	18.13 (6.23)		16.01 (6.94)	16.31 (7.31)	1.21		0.31

Mean values were calculated from the available data, with no imputation for missing values. The number of participants at each assessment time varied due to attrition at different stages of the study

**Fig. 3:**
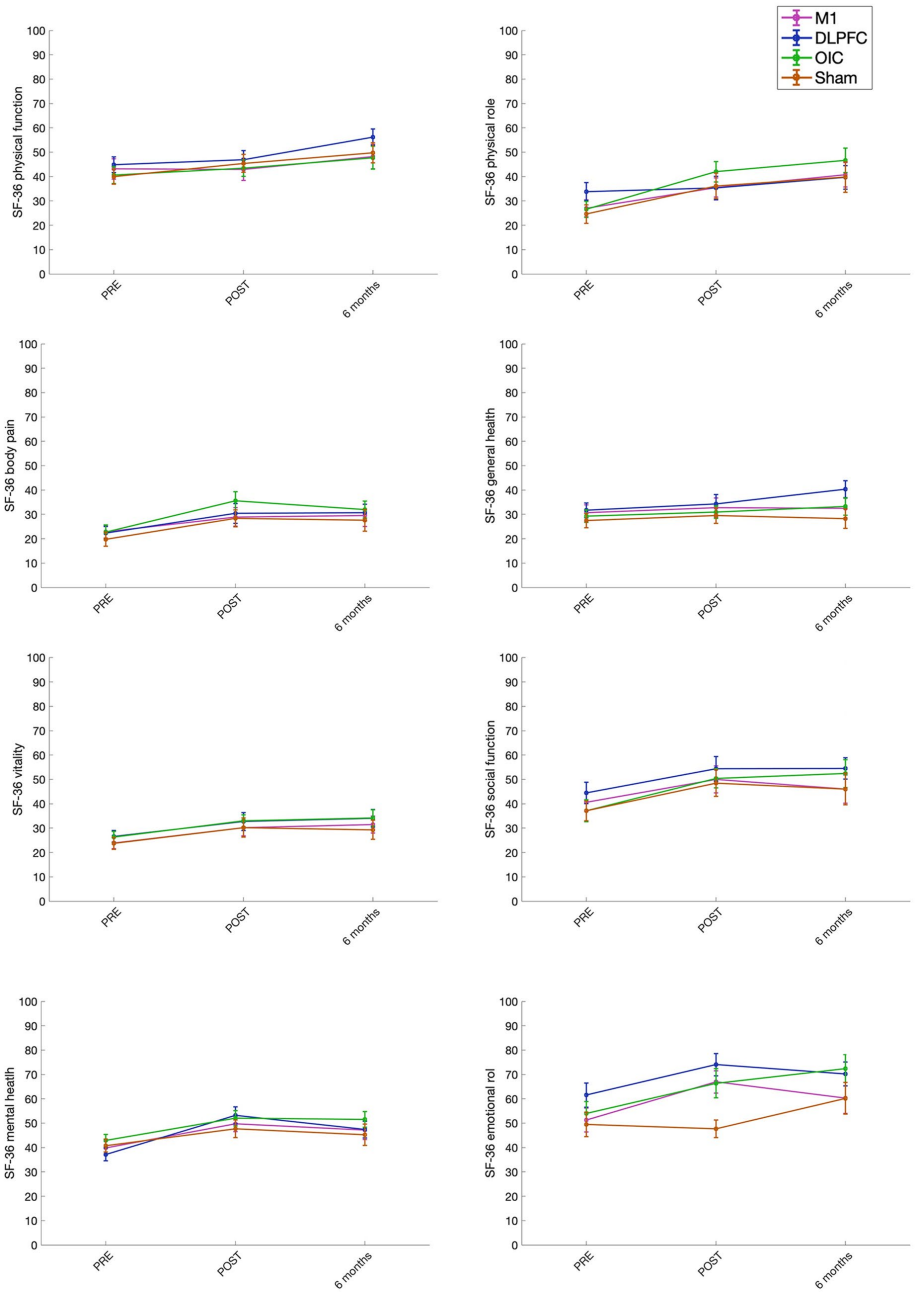
Comparison of pre-, post- and follow-up treatment assessment of the eight SF-36 subscales for the different tDCS stimulation groups (M1, DLPFC, OIC and Sham). Higher scores indicate improvement in QoL

**Fig. 4:**
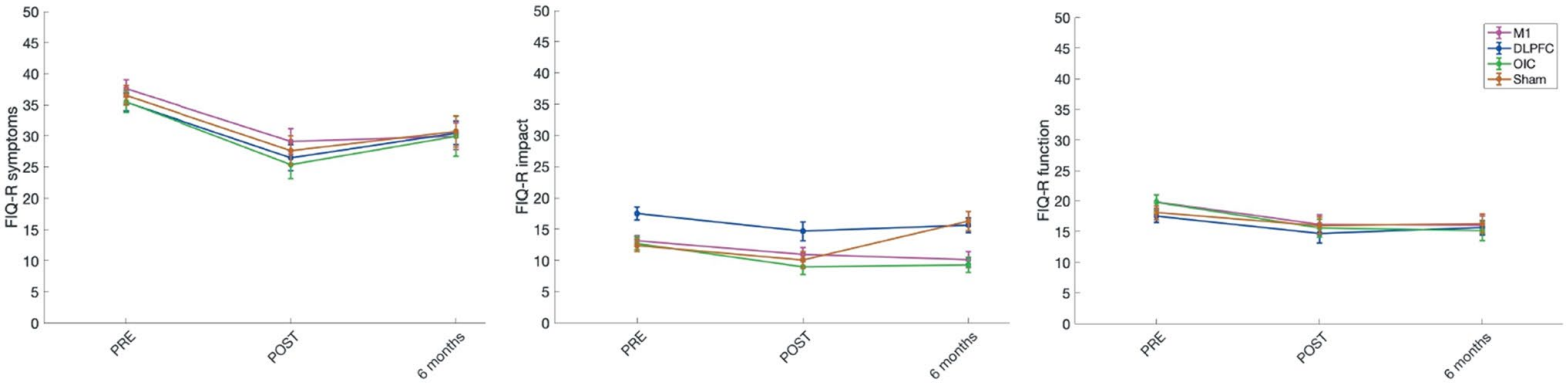
Comparison of pre-, post- and follow-up treatment assessment of the FIQ-R sub-scales for the different tDCS stimulation groups (M1, DLPFC, OIC and Sham). Lower scores represent improvement (i. e., decrease in symptom severity, impact, or functioning) in FM patients

The repeated-measures ANOVAs for SF-36 and FIQ-R (total scores and sub-scales) revealed a significant Time effect for all the selected outcome variables. Post hoc analysis showed significant differences between pre- and (immediately) post-treatment scores, and between pre- and 6-month treatment assessment, but not between post- and 6-month treatment assessment, for most of the variables (with large effect sizes (*ηp*^2^ > 0.14) for the variables *SF-36 mean, Emotional Role, FIQ-R total, FIQ-R Impact* and *FIQ-R Function;* and intermediate effect sizes (*ηp*^2^ > 0.06) for *Body Pain, Vitality,* and *Social Function)*. For other variables (*Physical role*, *Mental health,* and *FIQ-R symptoms)*, significant differences between all the time points (pre-treatment, post-treatment, and follow-up) were observed, with large effect sizes (*ηp*^2^ > 0.14). In *General Health*, there was no significant difference between pre- and post-treatment assessments; however, there was a significant difference between pre- and follow-up, with a small effect size effect (*ηp*^2^ > 0.01). There were no significant Group or Time*Group effects for any of the outcome variables (see Table 3, and Figs. 3 and 4). Therefore, QoL and FM impact on daily life improved in all the groups after tDCS (both, active and sham), and this change was maintained for at least 6 months.^1^

**Table 3:** Repeated measures ANOVA results for the clinical variables (with mean or median imputation for missing data) and size (*ηp*^2^) of the significant effects

ANOVA with imputation										
	Time effect			Group effect		Time*Group Effect		Post hoc analysis t(*p*)		
	*F*	*p*	*ηp*^2^	*F*	*P*	*F*	*p*	Pre vs post assessment	Pre vs 6-month follow-up assessment	Post vs 6-month follow-up assessment
SF-36 Mean	33.882	0.000	0.21	1.346	0.263	0.645	0.694	− 6.845 (0.000)	− 7.324 (0.000)	− 0.479 (1.000)
Physical Function	16.340	0.000	0.11	0.782	0.506	0.752	0.608	2.609 (0.047)	− 4.293 (0.000)	− 6.902 (0.000)
Physical Role	27.504	0.000	0.18	0.463	0.708	1.55	0.161	− 4.839 (0.000)	− 7.287 (0.000)	− 2.448 (0.008)
Body Pain	19.433	0.000	0.13	0.712	0.547	0.435	0.855	− 5.647 (0.000)	− 5.110 (0.000)	0.537 (0.610)
General Health	4.668	0.010	0.04	1.404	0.245	1.251	0.281	− 1.877 (0.063)	− 3.026 (0.007)	− 0.755 (0.193)
Vitality	15.629	0.000	0.11	0.761	0.518	0.035	1.000	− 4.500 (0.000)	− 5.123 (0.000)	− 0.623 (0.518)
Social Function	15.462	0.000	0.11	1.103	0.351	0.459	0.838	− 5.063 (0.000)	− 4.523 (0.000)	0.540 (0.591)
Mental Health	36.866	0.000	0.22	0.749	0.525	1.449	0.196	− 8.304 (0.000)	− 6.044 (0.000)	2.260 (0.017)
Emotional Role	22.302	0.000	0.15	1.807	0.149	0.957	0.455	− 6.191 (0.000)	− 5.265 (0.000)	0.926 (0.281)
FIQ-R total	49.906	0.000	0.28	0.504	0.680	0.515	0.797	9.274 (0.000)	7.855 (0.000)	− 1419 (0.128)
FIQ-R symptoms	60.97	0.000	0.32	0.411	0.745	0.569	0.755	10.869 (0.000)	7.123 (0.000)	− 3.746 (0.000)
FIQ-R Impact	22.376	0.000	0.15	1.053	0.372	0.443	0.850	5.247 (0.000)	6.217 (0.000)	0.970 (0.339)
FIQ-R function	23.157	0.000	0.15	0.447	0.720	1.001	0.425	6.089 (0.000)	5.677 (0.000)	− 0.413 (0.680)

In relation to the percentage of clinical improvement, all groups presented an improvement of more than 26% in the post-treatment and more than 29% in the follow-up at 6 months in the SF-36 total score. In the FIQ-R total score, all groups showed an improvement of more than 18% at post-treatment and more than 11% at follow-up. One-way ANOVAs showed no significant differences between groups in clinical improvement for any of the questionnaires (SF-36 and FIQ-R) at any time point (post-treatment and follow-up) (see Table 4).

**Table 4:** Mean values (Standard Deviation in parentheses) and one − way ANOVA results for the percentage of improvement of total scores of SF-36 and FIQ-R

	M1		DLPFC		OIC		Sham		One-way ANOVA			
	Pre vs Post	Pre vs Follow-up	Pre vs Post	Pre vs Follow-up	Pre vs Post	Pre vs Follow-up	Pre vs Post	Pre vs Follow-up	Pre vs Post		Pre vs Follow-up	
									*F*	*p*	*F*	*p*
SF-36 Mean	26.11 (40.99)	29.84 (50.97)	33.10 (55.42)	38.07 (51.76)	45.10 (88.57)	50.88 (76.57)	40.73 (85.37)	32.92 (81.11)	0.480	0.697	0.652	0.583
FIQ-R total	19.28 (29.04)	19.03 (26.38)	20.66 (26.90)	11.27 (26.66)	26.05 (28.09)	18.51 (36.64)	18.65 (26.74)	18.47 (28.59)	0.480	0.697	0.519	0.670

Comparisons between pre-treatment vs. post-treatment and pre-treatment vs. follow-up

## Discussion

Although tDCS has been established as effective for FM management [21], evidence of its effectiveness is not robust enough. The QoL is a widely recommended index of response to treatment [40, 41]. Although QoL is severely affected in patients with FM, most studies on tDCS efficacy have used self-reported pain levels as the main outcome variable [22]. Thus, to provide robust evidence on the effect of tDCS on FM, we oriented our study to determine how this technique affects the QoL of patients. We performed a double-blind sham-controlled clinical trial in a sample of 130 patients with FM, using 15 stimulation sessions and a 6-month follow-up. The findings showed that active tDCS (irrespectively of the stimulation target) is not better than sham tDCS for improving the QoL of the patients.

In the study, we used a general questionnaire (the SF-36) to cover all the dimensions of QoL [63] and a specific tool (the FIQ-R) to explore the functioning and impact of FM symptoms [60, 61]. The results obtained for all the subscales and variables analysed were very consistent and support the efficacy of the intervention in improving QoL, regardless of the treatment group. Using the SF-36, some studies also reported improvement in QoL after both sham and active tDCS, with no differences between groups when M1 [48, 64] or DLPFC [49] were stimulated. Nevertheless, there are also discrepant results on the effects of tDCS on QoL. Thus, some authors reported lack of effect [29] whilst others reported greater improvement in QoL after active than after sham tDCS, especially when delivered over M1 [49, 52]. Concerning the FIQ-R questionnaire and contrary to our results, most studies found a greater reduction of the impact of FM symptoms after active tDCS over M1 and DLPFC than after sham tDCS [31, 48, 49, 64]. The discrepancy between those studies and ours may be due to the stimulation protocol used and the sample size. In this regard, our study was performed using a large number of tDCS sessions, that enhances the effects of tDCS and its durability [31, 50], but also the unspecific effects [65]; with double-blind control, to ensure that the effects of treatment are not overestimated [66]; and in the larger sample to date, what adds robustness to the results obtained about tDCS effectiveness.

Another major strength of this study is that we conducted a longer (6-month) follow-up than used in previous similar studies. We found that the clinical improvement in QoL persisted 6 months after treatment, with no differences between groups. Previous studies have reported that the effects of tDCS over M1 can last up to fifteen days for pain and depression symptoms [50], 1 month for the impact of FM assessed by the FIQ [49], and 2 months for clinical pain [31].

An additional aim of the present study was to identify the optimal target for tDCS as for the positive effects on QoL. It has been suggested that stimulation over M1 may reduce pain due to the connections with thalamus, brainstem, cingulate gyrus, prefrontal cortex and insula [26, 67–69], although the precise neurophysiological mechanisms are not fully understood. Moreover, the DLPFC is an important area involved in the cognitive processing of pain [28, 70–72]. tDCS over the left DLPFC has been associated with control of cognitive aspects of pain in patients with FM [73]. However, we hypothesized that stimulation over a specific area related to pain that presumably plays a crucial role in FM [74–76], such as the operculo-insular cortex (OIC), would be more effective than stimulation of the traditional and less specific targets (M1 and DLPFC). Contrary to the expectations, comparison of the effects of stimulation of these three areas did not reveal any of them as a superior target.

To really understand the clinical significance of the improvements found, we calculated the minimal clinically important difference (MCID) and found that all the groups improved in SF-36 scores more than 26% immediately after treatment and more than 29% at follow-up, without differences between groups. For patients with osteoarthritis, previous research reported MCID for SF-36 between 10 and 12% [77, 78], whilst to our knowledge no data for patients with FM are available. In FIQ-R, we found improvements superior to 18% immediately after treatment and to 11% in the follow-up. There is no consensus about what is the MCID for FIQ. Although recent literature suggests 45.5% improvement in FIQ-R score as the MCID [79], most previous studies using the FIQ established it as 14% [80, 81]. Since the validation analysis and psychometric properties of the FIQ-R total score have shown a strong correlation with the FIQ ones, the relative positions of patients on the two scales are considered to be very similar [60]. Thus, considering the 14% cut-off score, we could conclude that a clinically significant improvement in the global impact of FM was observed immediately after treatment but not after 6 months of follow-up. Again, the improvements were observed in all the groups, even after sham stimulation.

Considering the overall results, the improvement in QoL due to the intervention can be mainly attributed to a placebo effect rather than to the tDCS itself. In outcomes such as pain, the magnitude of change in the placebo arm is large and long-lasting (explaining about 80% of the improvement in the active arm) [82]. In pain conditions, placebo has been positively related to large sample sizes (by the motivation and expectations of being part of a rigorous, professional and well-funded study) and with long duration trials (by a positive feedback mechanism: initially perceived pain relief leads to increased analgesia throughout the trial) [65]. The presence of the placebo effect in tDCS treatments has also been widely observed [83], probably due to the positive expectations that patients have concerning this novel intervention. Moreover, we found that the placebo effect on QoL lasted for 6 months. Similarly, it has been observed that the placebo response could relieve pain at least during 3 months, followed by stabilization without reversal [65]. This has been explained by a process of conditioning. A conditioned response needs a reward (reinforcement) to be maintained for long periods; thus, the analgesia obtained with the placebo matches the individual's expectations and predictions, and the pain relief achieved can induce a reward sensation, which is itself analgesic, thereby sustaining a positive feedback loop and maintaining pain reduction for a long period [84]. Regarding the neurobiological basis, it has been suggested that placebo stimulation may activate one of the main analgesic mechanisms: the endogenous μ-opioid receptor-mediated neurotransmission allocated in periaqueductal grey matter (PAG), precuneus, and thalamus [85]. Moreover, it has been found than before applying tDCS (active or sham) there is an early placebo effect (activation of this μ-opioid neurotransmission) that is correlated with endogenous μ-opioid receptors activation during active tDCS [85]. Thus, according to this preliminary finding, the success of M1 tDCS analgesia could depend on the individual susceptibility to mobilize μ-opioid activity related to placebo. Nevertheless, more research is needed to fully understand the neurobiological basis of the placebo effect.

The above results should be interpreted in the light of a number of limitations. First, the FIQ-R does not provide a complete profile of the functioning of patients with FM; thus, future research should include specific indices of functioning such as the WHODAS 2.0 [86]. Second, the design of our study may make it difficult to differentiate between the effect of tDCS itself and the effect of the intervention. In this vein, previous studies found that both sample size and trial duration (i.e., number of face-to-face visits) were significantly associated with placebo response magnitude [65, 87, 88]. Several unspecific variables may be influencing this relation, since the 15 session patient-to-patient contact probably increased social support and peer understanding, which are crucial for promoting health improvement [89]. Also, the daily commute to the health centre during 3 weeks could have a positive impact on the physical (exercising) and emotional health (getting out of the house, distraction from daily routine…) of the patients. Moreover, the therapists had a strong commitment and empathy with the participants. All of this could lead to the Hawthorne effect, understood as a change in participants’ behaviour as a motivational response to the interest, care or attention received through observation and assessment, which is influenced by the researchers wishes [90, 91]. This effect has been widely observed in research on pain treatment [91, 92]; in fact, it has been reported that the treatment effect observed in some clinical trials may be upwardly biased due to the Hawthorne effect [93]. Finally, although the sample size is larger than in previous studies in the field, it may not have been large enough to detect group effects of small size. In order to address such non-specific effects of the intervention, we consider that home-based treatments may be a promising alternative for evaluating the effects of tDCS (or other modulation techniques) on the quality of life of patients with FM. Applying tDCS at home may minimize the effect of social interaction (with the therapist and other patients), reduce intervention costs and allow the inclusion of a larger number of participants. Furthermore, to increase knowledge about tDCS and the sham-placebo effect in clinical trials, it would be interesting to conduct tDCS clinical trials evaluating its effect on brain activity assessed with neuroimaging techniques and adding an untreated group to the experimental design to assess the effects more accurately.

### Conclusions

Here we assessed the efficacy of different tDCS cortical targets to improve QoL. We found that fifteen sessions of tDCS, irrespective of cortical target and active/simulated condition, improved patients' well-being by achieving a clinically significant improvement (measured immediately after the end of treatment and 6 months later). The observed improvement may be explained by a placebo effect probably related to the positive expectations on the efficacy of neuromodulation techniques and to non-specific psychosocial variables.

## Supplementary Information

Below is the link to the electronic supplementary material.

Supplementary file1 (DOCX 27 KB)

## Data Availability

The authors confirm that the data supporting the findings of this study are available from the corresponding author NS-V on request.

## Footnotes

1 In general, the results with imputation were similar to those found in the analysis without imputation, except for the physical function variable, where we did not find any significant effect (see Table S2; supplementary material).
