# Correction to: Active and sham transcranial direct current stimulation (tDCS) improved quality of life in female patients with fibromyalgia

**DOI:** 10.1007/s11136-023-03478-y

**Published:** 2023-07-24

**Authors:** N. Samartin-Veiga, A. J. González-Villar, M. Pidal-Miranda, A. Vázquez-Millán, M. T. Carrillo-de-la-Peña

**Affiliations:** 1 Brain and Pain (BaP) Lab, Departamento de Psicoloxía Clínica y Psicobioloxía, Facultade de Psicoloxia, Universidade de Santiago de Compostela, Campus Vida, 15782 Santiago de Compostela, A Coruña, Spain; 2 Psychological Neuroscience Lab, Research Center in Psychology, School of Psychology, University of Minho, Braga, Portugal

**Correction to: Quality of Life Research (2022) 31:2519–2534** 10.1007/s11136-022-03106-1

In the published version of the manuscript, reference was made to a paper as “Samartin-Veiga et al. (2022)” and no corresponding bibliographic information was included.

This omitted reference refers to Samartin-Veiga, N., Pidal-Miranda, M., González-Villar, A. J., Bradley, C., Garcia-Larrea, L., O'Brien, A. T., & Carrillo-de-la-Peña, M. T. (2022). Transcranial direct current stimulation of 3 cortical targets is no more effective than placebo as treatment for fibromyalgia: A double-blind sham-controlled clinical trial. *Pain*, *163*(7), e850–e861. 10.1097/j.pain.0000000000002493”.

