# Supplementary material for: Active and sham transcranial direct current stimulation (tDCS) improved quality of life in female patients with fibromyalgia
Source: Qual Life Res. 2022 Mar 1;31(8):2519–34. doi: 10.1007/s11136-022-03106-1 (PMC9250466; doi:10.1007/s11136-022-03106-1)
Supplement: Supplementary file 1 — Supplementary file1 (DOCX 27 KB) [file 11136_2022_3106_MOESM1_ESM.docx]

**Table S1.** Model comparison test of LMR model variants for clinical outcomes (total and subscales scores of SF-36 and FIQ-R). AIC model selection (lowest AIC, best fit to data)^[[1]](#footnote-1)^.

| **LMR Models SF-36 Mean** | **AIC** | **BIC** | **logLik** |
| --- | --- | --- | --- |
| LOCFB imputation | 3148,9 | 3212,3 | -1558,5 |
| Mean imputation | 3054,6 | 3118,0 | -1511,3 |
| Median imputation | 3053,4 | 3116,8 | -1510,7 |
| **LMR Models Physical Function** | **AIC** | **BIC** | **logLik** |
| LOCFB imputation | 3331,8 | 3395,1 | -1649,9 |
| Mean imputation | 3255,8 | 3319,1 | -1611,9 |
| Median imputation | 3259,6 | 3323,0 | -1613,8 |
| **LMR Models Physical Role** | **AIC** | **BIC** | **logLik** |
| LOCFB imputation | 3480,1 | 3543,4 | -1724,0 |
| Mean imputation | 3391,6 | 3454,9 | -1679,8 |
| Median imputation | 3394,2 | 3457,6 | -1681,1 |
| **LMR Models Body Pain** | **AIC** | **BIC** | **logLik** |
| LOCFB imputation | 3324,8 | 3388,2 | -1646,4 |
| Mean imputation | 3242,9 | 3306,2 | -1605,4 |
| Median imputation | 3246,2 | 3309,5 | -1607,1 |
| **LMR Models General Health** | **AIC** | **BIC** | **logLik** |
| LOCFB imputation | 3213,4 | 3276,7 | -1590,7 |
| Mean imputation | 3120,6 | 3183,9 | -1544,3 |
| Median imputation | 3120,6 | 3183,9 | -1544,3 |
| **LMR Models Vitality** | **AIC** | **BIC** | **logLik** |
| LOCFB imputation | 3218,8 | 3282,2 | -1593,4 |
| Mean imputation | 3136,1 | 3199,4 | -1552,0 |
| Median imputation | 3137,2 | 3200,5 | -1552,6 |
| **LMR Models Social Function** | **AIC** | **BIC** | **logLik** |
| LOCFB imputation | 3556,5 | 3619,9 | -1762,3 |
| Mean imputation | 3503,5 | 3566,9 | -1735,8 |
| Median imputation | 3509,2 | 3572,6 | -1738,6 |
| **LMR Models Mental Health** | **AIC** | **BIC** | **logLik** |
| LOCFB imputation | 3197,9 | 3261,3 | -1583,0 |
| Mean imputation | 3119,2 | 3182,6 | -1543,6 |
| Median imputation | 3122,2 | 3185,5 | -1545,1 |
| **LMR Models Emotional Role** | **AIC** | **BIC** | **logLik** |
| LOCFB imputation | 3607,9 | 3671,3 | -1788,0 |
| Mean imputation | 3536,2 | 3599,6 | -1752,1 |
| Median imputation | 3540,5 | 3603,9 | -1754,3 |
| **LMR Models FIQ-R Total** | **AIC** | **BIC** | **logLik** |
| LOCFB imputation | 3399,8 | 3463,1 | -1683,9 |
| Mean imputation | 3308,6 | 3371,9 | -1638,3 |
| Median imputation | 3309,0 | 3372,3 | -1638,5 |
| **LMR Models FIQ-R Symptoms** | **AIC** | **BIC** | **logLik** |
| LOCFB imputation | 2873,5 | 2936,8 | -1420,8 |
| Mean imputation | 2787,1 | 2850,5 | -1377,6 |
| Median imputation | 2789,9 | 2853,2 | -1378,9 |
| **LMR Models FIQ-R Impact** | **AIC** | **BIC** | **logLik** |
| LOCFB imputation | 2426,1 | 2489,4 | -1197,0 |
| Mean imputation | 2347,6 | 2410,9 | -1157,8 |
| Median imputation | 2350,3 | 2413,7 | -1159,2 |
| **Modelos LMR para FIQ-R Function** | **AIC** | **BIC** | **logLik** |
| LOCFB imputation | 2551,4 | 2614,7 | -1259,7 |
| Mean imputation | 2447,8 | 2511,2 | -1207,9 |
| Median imputation | 2448,6 | 2511,9 | -1208,3 |

# *AIC: Akaike information criterion; BIC: Bayesian information criterion; LOCFB: Last Observation Carried Forward and Backward; SF-36: Short Form Health Survey; FIQ: Fibromyalgia Impact Questionnaire.*

**Table S2**. Repeated-measures ANOVAs without imputation of missing data for SF-36 and FIQ-R (total scores and sub-scales). These analyses showed a significant Time effect for all the variables (except for the SF-36 subscale Physical Function). There was no significant Group or Time*Group effect for any of the outcome variables. Therefore, all the groups improved in QoL and functioning after tDCS, and this improvement was maintained during six months.

| ANOVA without imputation | | | | | | | |
| --- | --- | --- | --- | --- | --- | --- | --- |
|  | Time effect | | | Group effect | | Time*Group  effect | |
|  | F | p | ηp² | F | p | F | p |
| SF-36 Mean | 20.53 | 0.00 | 0.19 | 0.73 | 0.54 | 0.43 | 0.86 |
| Physical Function | 1.18 | 0.31 | - | 0.03 | 0.99 | 0.74 | 0.62 |
| Physical Role | 18.58 | 0.00 | 0.17 | 0.22 | 0.88 | 0.63 | 0.71 |
| Body Pain | 59.48 | 0.00 | 0.39 | 0.25 | 0.87 | 0.40 | 0.88 |
| General Health | 8.91 | 0.00 | 0.09 | 1.12 | 0.34 | 0.84 | 0.54 |
| Vitality | 7.02 | 0.00 | 0.07 | 0.14 | 0.99 | 0.32 | 0.81 |
| Social Function | 8.60 | 0.00 | 0.09 | 0.21 | 0.89 | 0.15 | 0.99 |
| Mental Health | 119.18 | 0.00 | 0.54 | 0.50 | 0.69 | 0.40 | 0.88 |
| Emotional Role | 14.08 | 0.00 | 0.13 | 0.95 | 0.42 | 0.94 | 0.47 |
| FIQ-R total | 28.19 | 0.00 | 0.23 | 0.19 | 0.90 | 0.12 | 0.99 |
| FIQ-R Symptoms | 33.71 | 0.00 | 0.30 | 0.22 | 0.88 | 0.30 | 0.94 |
| FIQ-R Impact | 11.43 | 0.00 | 0.11 | 0.27 | 0.85 | 0.29 | 0.94 |
| FIQ-R Function | 12.00 | 0.00 | 0.11 | 0.15 | 0.93 | 0.60 | 0.73 |

1. In the linear mixed regression (LMR) models tested, the fixed effect was the interaction between group randomization and time (treatment points), and the random effects were the subject-level intercepts with random slopes over time. For QoL measurements (SF-36 and FIQ-R scales and subscales), time was categorized into pre-treatment, post-treatment, and 6 months follow-up. Analyses were performed using the R packages lme4 and emmeans (R version 4.0.2, The R Foundation). [↑](#footnote-ref-1)
